# Analysis of the Stockholm Public Health Cohort: Exploring How Ultraviolet Radiation and Other Factors Associate with Skin Cancer

**DOI:** 10.1155/2024/7142055

**Published:** 2024-10-03

**Authors:** Lina U. Ivert, Henrik Dal, Ylva Rodvall, Bernt Lindelöf

**Affiliations:** ^1^ Dermatology and Venereology Unit Department of Medicine Solna Karolinska Institutet, Solna, Sweden; ^2^ Department of Dermatology Karolinska University Hospital Solna, Solna, Sweden; ^3^ Department of Global Public Health Karolinska Institutet, Solna, Sweden; ^4^ Centre for Epidemiology and Community Medicine, Region Stockholm, Solna, Sweden; ^5^ Theme Cancer Karolinska University Hospital, Solna, Sweden

## Abstract

**Objective:**

The primary aims of the study were to (1) explore the association of skin cancer and four ultraviolet radiation (UVR) indicators (sunbed use, healthcare data on diagnosed melanocytic nevi (MN) and actinic keratosis (AK), and latitude of birthplace), and (2) find factors other than UVR that could explain the increasing trend in incidence of skin cancers, including basal cell carcinoma (BCC), squamous cell carcinoma (SCC), and cutaneous malignant melanoma (CMM).

**Methods:**

This population-based cohort study used self-reported questionnaire data from the Stockholm Public Health Cohort, encompassing 103 questions, merged with data from Swedish national registers. The study population included almost 35,000 Swedish-born people aged 30–66 years in 2014. Binomial logistic regression was employed for analysis. A forward stepwise regression was applied to select significant risk factors among all the factors included. We tentatively tested >30 variables separately for any relationship with each of the three skin cancers. A 5% level of significance was applied. Melanoma in situ and SCC in situ were excluded.

**Results:**

The four UVR-related factors (sunbed use, being diagnosed with AK or MN, birthplace latitude) had a significant association with at least one of the three skin cancers that remained after adjustment including behavioural, social, hereditary, and medical factors. Sunbed use >10 times before age 30 years was related to all three skin cancers; SCC adjusted odds ratio (aOR) 1.66, 95% confidence interval (CI) 1.12–2.47, CMM (aOR 1.57, 95% CI 1.11–2.22), and the clearest dose-response association with BCC (aOR 1.74, 95% CI 1.46–2.06). None of the examined lifestyle factors, except physical activity, had any significant associations with UVR indicators or skin cancer.

**Conclusion:**

We did not find any preventable explanatory cause other than UVR exposure for the increasing incidence of skin cancers. This result remained when adjusting for an array of possible confounders including behavioural, social, hereditary, and medical factors.

## 1. Introduction

In Sweden, the three main types of skin cancer, basal cell carcinoma (BCC), squamous cell carcinoma (SCC), and cutaneous malignant melanoma (CMM), all rank among the ten most common cancers and are thus a major public health concern. Incidence rates for BCC were 515 and 513/100,000 (year 2011) for males and females, respectively, for SCC 83 and 61/100,000 (year 2016), and for CMM 43 and 40/100,000 (year 2016), with melanoma in situ and SCC in situ excluded [1]. Sunburns and accumulated sun exposure are established risk factors for skin cancer, the first for CMM and the latter for SCC in particular [2, 3]. Both intermittent and accumulated sun exposure seem to be involved in BCC [4]. Additionally, sunbed use, particularly before age 30 years, is an established risk factor for skin cancer [5, 6]. Both CMM and SCC have had a steady incidence increase since the early 1970s, even though the protective ozone layer over Sweden has been unchanged since the 1950s [7, 8]. The increase in skin cancers thus appears to be explained by changed sun habits. Studies have shown how the shift of outdoor to indoor work has impacted the occurrence of both SCC and CMM, and their locations on the body, over several decades [9, 10]. The relationships between skin cancer and occupation, lifestyle, and socioeconomic factors have been studied to a lesser extent [11]. However, it is known that professionals in the affluent segments of the Swedish population run a higher risk of BCC, which is believed to be related to lifestyle, for instance through leisure-time exposure [12]. Registration of BCC occurrence is relatively recent, so its historical trends are unknown.

Sunbeds emitting ultraviolet radiation (UVR) have existed for only a few decades, but their use is widespread. Such use may exacerbate preexisting actinic keratoses (AK) and melanocytic nevi (MN), predictors or precursors of SCC and CMM, respectively [13, 14]. According to a health environmental report from 2017, 20% of people aged 15–29 years had used a sunbed in the preceding 12 months [15].

Many factors potentially contributing to the increase in skin cancer incidence—and the interplay between them—remain sparsely studied. They include an array of behavioural, social, and medical factors. The two primary aims of this study were (1) to explore the association of skin cancer and four UVR indicators (sunbed use, healthcare data on diagnosed MN and AK, and latitude of birthplace) with a particular focus on sunbed use and (2) to see if we could find non-UVR factors that could explain the long-term increasing trend in skin cancer incidence.

## 2. Materials and Methods

### 2.1. Study Design, Population, and Databases

The data used originate from the Stockholm Public Health Cohort (SPHC) [16], a population-based cohort study with self-reported questionnaire data, and from Swedish national registers. The study population was in the SPHC and encompassed thousands of randomly selected people living in Stockholm County in 2014 who joined the cohort study by answering a postal/Internet questionnaire (including 103 questions) in that year. Two selection criteria—Swedish-born and age 30–66 years (in 2014)—were applied to enable matching of the study population with the commercial introduction of cosmetic sunbeds in Sweden in 1978. The criterion of Swedish-born was used to take some account of eye and hair colour as well as skin type. To enable a complete follow-up, only participants who had never emigrated and who alive at the end of the study were included. A total of 44,750 respondents aged 30–66 years participated in the survey in 2014 and delivered valid answers to the question on sunbed use. Out of those, 9,909 respondents (22%) were excluded from analysis, with 7,411 (16.6%) excluded due to foreign origin. The 2014 questionnaire contained one question about sunbed use before age 30 years, which is of particular importance in this study. The questionnaire covered self-rated health, diagnosed diseases, social factors, and lifestyle.

The cohort was linked to several national registers, using anonymized identity numbers based on personal identity numbers. Four registers from the Board of Health and Welfare were used: the Swedish Cancer Registry (SCR) (years 1958–2017), the Swedish Basal Carcinoma Register (BCR) (2004–2018), the National Patient Register (NPR) with in- and outpatient care (2001–2018), and the Swedish Cause of Death Register (1952–2017). From Statistics Sweden, we used the Register of Total Population, the Multigenerational Register (to retrieve data on parental cancer), the Register of Migrations, and the Longitudinal Integrated Database for Health Insurance and Labor Market Studies (2001–2018). Written consent was obtained from all participants.

### 2.2. Ethics

The study was approved by the Swedish Ethical Review Authority (#2019-02457).

### 2.3. Skin Cancer Outcomes

The three skin cancer outcomes were captured from the BCR and the SCR. BCC was defined as any event in the BCR, SCC was defined as code 191XX (ICD-7), benign or malignant, in the SCR, and CMM as code 190XX (ICD-7), only malignant, in the SCR. Melanoma in situ and SCC in situ were excluded.

### 2.4. Covariates

Potential confounders of skin cancer risk from UVR were analysed vs. four UVR-related independent variables: sunbed use < age 30 years, being diagnosed with AK (ICD-10 L57XX) or MN (ICD-10 D22XX), and latitude of birthplace. In order to have sunbed use and birthplace as binary outcomes in Table 1, they were dichotomized (sunbed use age <30 years, 0 times = 0, more than once = 1, sunbed use 1 time eliminated from analysis, birthplace in the southernmost region = 0, birthplace further north = 1). MN was used as proxy for UVR exposure as body sites with high nevus count have been associated with UVR exposure [17, 18]. Information about the number and anatomic distribution of MN or AK were not available, only the diagnoses. We assumed that patients with a high number of nevi or several actinic keratoses more often tend to seek care and get diagnosed with MN/AK. Age and sex relate to differences in UVR exposure, lifestyle, and immune defence. Lastly, lifestyle factors in terms of smoking, alcohol consumption, dietary habits and physical activity, and social factors (education level, type of housing) that could impact skin cancer risk were taken into account.

We combined height and weight into the variable “body shape” to get an indicator of differences between responders in skin cell number. Furthermore, obesity is an established risk factor for several cancer types. Some skin diagnoses that might prompt participants to seek or avoid UVR exposure was retrieved, e.g., eczema, vitiligo, psoriasis and rosacea. Seborrheic keratosis was included as it can be a sign of extrinsic aging, particularly due to chronic UVR exposure [19]. Virus warts were used as a proxy for human papillomavirus (HPV) infection. Diabetes was included, as it has been associated with more aggressive cutaneous melanoma [20]. Organ transplants, immune system disorders (ICD-10 D80–D89), and influenza vaccine (during the preceding 12 months) was used as a proxy for poor immune defence. The last would generally indicate preexisting health conditions, as the participants were aged ≤66 years. Variables are presented in detail in Supplement Table I. In the final skin cancer analysis, shown in Table 2, we included only variables with a significant relationship to at least one of the UVR indicators in Table 1. Two of the UVR-related variables are considered both as indicators of previous UVR exposure and as possible intermediate stages of cancer [21, 22] and commonly registered in the same patients: (1) AK and SCC and (2) MN and CMM. The odds ratios (ORs) were not calculated as the intermediate variables would bias our estimates.

### 2.5. Missing Values

The 2014 questionnaire had missing values on all questions, ranging from 0.7% on a question of diabetes at the beginning of the questionnaire, 2.7% on the question about sunbed use, around 3% on questions about physical activity, and >4% on questions on dietary habits with a subset of 14 response categories, which may have been experienced as cumbersome. Further, 0.4% of the participants' mothers and 1.2% of the participants' fathers were unknown. When all variables were analysed together, over 10% of the population had at least one item missing. Instead of excluding all these participants, we created a category of “missing value” for all variables and included this category in the analysis.

### 2.6. Statistical Analysis

Binomial logistic regression was employed for analysis. A forward stepwise regression was applied to select significant risk factors among all the factors included. We tentatively tested >30 variables separately for any relationship with each of the three skin cancers. Sensitivity analyses were run to check if the answers to the sunbed question were distorted by respondents' skin cancer. Here, only skin cancers registered after the questionnaire were included, to check if the results of sunbed data were the same. We also did a quality check by running the same analysis as that underlying the data in Table 1, excluding cases with missing values, to check if the results for sunbed use were the same. Since we were able to find rational explanations for all significant associations, we have not made adjustments for potential errors due to multiple comparisons. A 5% level of significance was applied, and IBM SPSS version 26 was used for all analyses. A higher bivariate association corresponds to a higher percentage and often a lower mean age at first incidence of cancer.

## 3. Results

Among participants in the SPHC who completed the questionnaire, over 1,100 individuals were diagnosed with BCC during the period from 2004 to 2018, and more than 200 individuals were diagnosed with either SCC or CMM. The UVR indicators were even more common, with thousands of sunbed users and patients diagnosed with MN and AK (Table 3).

### 3.1. Sunbed Use

Sunbed use before age 30 years was significantly associated with female sex (Table 1), being diagnosed with MN, AK, and being born in north (Supplement Table II). We also found an association between sunbed use and lower physical activity, higher frequency of binge drinking, and average education levels (Table 1).

### 3.2. Skin Cancer

The prevalence of CMM was 1.0% in persons born in south Sweden, compared with 0.5% of persons born in north Sweden (Table 2). Females had a lower risk of BCC in the studied age span. Age had a significant positive association with all cancers.

### 3.3. Lifestyle, Social, and Physical Factors vs. MN, AK, and Skin Cancer Risk

Table 1 shows that higher education was associated with MN and AK. Moreover, AK was associated with physical activity. In overweight individuals of different heights, an inverse association was observed with diagnosed MN and AK compared to the reference category: short, normal weight individuals. As shown in Table 2, height was associated with SCC among people with normal weight. Short, overweight people had a lower occurrence of BCC than short, normal weight people. People who never binge drank alcohol had a higher OR for CMM compared with those who sometimes binge drank. None of the other examined lifestyle factors, except physical activity, had any significant associations with UVR indicators or skin cancer. Data on smoking and the consumption of vegetables and fruits are not shown; however, these factors were not associated with skin cancer. Additionally, the type of housing and level of education were not found to be associated with skin cancer (Supplement Table III).

### 3.4. UVR-Related Factors (UVR Indicators) vs. Skin Cancer Risk

As shown in Table 2, the four UVR-related factors (sunbed use, being diagnosed with AK or MN, birthplace latitude) had a significant association with at least one of the three skin cancers. Being diagnosed with AK more than trebled the odds of BCC (both sexes), but was also associated with CMM. Diagnosed MN was found to double the odds of BCC and was associated with SCC. Sunbed use >10 times before age 30 years increased the odds of all skin cancers. For sunbed use, the strongest dose-response association was with BCC.

### 3.5. Previous Skin Cancer and Heredity vs. Skin Cancer Risk

Table 2 shows that a past diagnosis of BCC greatly increased the risk of SCC and CMM and vice versa. The type of parental skin cancer was shown to predict the type of skin cancer that affected the offspring. Parental skin cancer was associated with one or more of the four UVR indicators (Supplement Table II).

### 3.6. Medical Conditions vs. Skin Cancer Risk

All skin diseases except psoriasis and vitiligo had a significant association with a UVR indicator and/or a skin cancer; the association between rosacea and SCC was particularly pronounced (Tables 1 and 2). Diabetes had a negative association with sunbed use, AK and BCC (Tables 1 and 2).

## 4. Discussion

In this cohort study, the variables related to UVR exposure (sunbed use, birthplace latitude, being diagnosed with AK or MN) were all clearly associated with at least one of the three skin cancers. Age was, unsurprisingly, a significant risk factor for all three skin cancers. If parents had skin cancer, study subjects had an increased risk of both skin cancer and UVR exposure measured with the selected UVR indicators.

The study results are in line with previous robust studies where sunbed use increased the risks of melanoma [23, 24] and nonmelanoma skin cancers [25]. The effect of having used a sunbed only once before age 30 years is unclear, sometimes displaying no association and sometimes associated with a higher risk of skin cancer compared with that of never-users. A strong skin reaction to sunbed use might deter further UVR exposure. Previous Swedish studies have shown strong associations between certain risk behaviours, e.g., sunbed use and smoking, among adolescents but not among adults [26, 27]. However, we found that sunbed use among adults could be associated with lifestyle factors such as less physical activity and high alcohol consumption. People who never binge drank alcohol had a higher OR for CMM compared with those who sometimes binge drank. We propose that this unexpected association is due to extreme age differences in both binge-drinking habits and the risk of CMM.

The finding that being born in south Sweden increased the risk only for CMM may be a result of sunburn and/or formation of nevi in childhood, and is congruent with previous studies [28]. Whiteman et al. have estimated that the occurrence of CMM will rise substantially by 2031 in Sweden and some other countries, probably due to high UVR exposure in the past and the aging population [29]. However, a previous study demonstrated positive results, showing that the number of MN decreased among 7-year-olds in Sweden between 2002 and 2009 [18]. A corresponding questionnaire administrated to their parents indicated improved sun protection behaviour. This hopefully confirms a current trend of increased awareness regarding the risks of UVR exposure.

Diabetes seems to have a negative association with BCC. Some studies have found an antineoplastic effect of antidiabetic medication, but this association needs further exploration [30]. We also found an association between rosacea and SCC congruent with a recent Danish cohort study. The authors suggested that rosacea might present as more serious in case of a history of high UVR exposure [31]. Interestingly, Rosacea was associated with all UVR indicators in the current study.

AK seems to be a marker of healthy lifestyle, excepting UVR exposure. In terms of diet and physical exercise, many individuals with otherwise healthy lifestyles frequently pursue physically demanding activities entailing high UVR exposure (sailing, golfing, alpine skiing, marathon races, etc.). We also suggest that the association between height and SCC in this study is related to skin type and sun behaviours. Sweden had a high frequency of tall people compared with other regions of birth (data not shown). Moreover, as seen in Table 1, people with higher education who own their own homes get more AK diagnoses, indicating that socioeconomic and behavioural factors indeed play an important role in UVR exposure. Some previous studies have discussed hormonal and genetic factors in relation to an association between height and skin cancer [32]. We identified a lower risk of BCC in a subgroup of overweight people. A link between higher BMI and lower risk of BCC has previously been shown—the mechanism behind this remains unclear, with both behavioural and biological patterns being suggested [33].

### 4.1. Strengths

This study based on extensive questionnaire data in a large sample, combined with established registers with high standards regarding coverage and verification, such as the NPR and the SCR, has a unique potential to explore some seemingly unrelated factors. Questionnaire data on socioeconomic factors and factors directly or indirectly associated with UVR exposure could be analysed. The study comprised almost 35,000 individuals. We did tests excluding responders who had cancer before the survey, but this did not change the results.

### 4.2. Limitations

As registration of BCC started only in January 2004, we estimate that around 200 people (16%) had undiagnosed BCC in this study. Most of the cancers affected our comparison group, which means that our risk estimates for BCC are a bit low. The response rates in the four waves of the cohort survey declined from 62% in 2002 to 38% in 2014. Adding to the initial nonresponse, respondents dropped off between the initial questionnaire and the 2014 questionnaire, when the question on sunbed use was included. Nonresponse was highest among males, younger people, people born outside of Sweden (though not included in this analysis) and people with low income and education. With a focus of interest upon the associations between various factors and skin cancer rather than prevalence, we considered this analysis to be relevant, and the findings (adjusted for sex, age, and socioeconomic factors) are in line with previous research.

We do not have a direct measure of outdoor UVR exposure. It could be that our result estimates are confounded by this major cause of skin cancer. However, a similar study adjusting for outdoor UVR and one review gives skin cancer risk estimates for sunbed use in line with our results [34, 35]. A German article found only medium associations between indoor and outdoor tanning [36]. The measures of UVR exposure, especially considering AK and MN, were imperfect, which could allow residual confounding. Unfortunately, information about the location and number of nevi was not available. We also acknowledge that the development and characteristics of nevi are influenced by genetic factors, along with environmental factors like sun exposure. This is an additional limitation when using MN as a marker of UVR index.

The associations between several common skin disorders, including skin cancer and MN and AK, as well as the association between seborrheic keratosis and skin cancers, may be affected by surveillance bias. Furthermore, seborrheic keratosis is considered a sign of general skin aging and extrinsic aging, particularly due to UVR exposure; however, other factors beyond UVR can also play a role [19]. We included warts as a proxy for HPV infection due to the association between HPV and nonmelanoma skin cancer [37]. We acknowledge the limitation that not all viral warts may share the same HPV type as those found in skin cancers, and the presence of HPV in nongenital lesions does not necessarily confirm its role as a pathogen. Psoriasis was collected from a survey question covering the entire lifespan, whereas other skin diagnoses were collected from the NPR for the years 2001–2018. This may explain why psoriasis was not associated with MN or AK, especially when accounting for surveillance bias. Lastly, NPR does not include primary care, which might have led to an underestimated prevalence of the common skin disorders managed in primary care.

## 5. Conclusion

We did not find any preventable explanatory cause other than UVR exposure for the increasing incidence of skin cancers. This result remained when adjusting for an array of possible confounders including behavioural, social, hereditary and medical factors. One example of the contributing factors identified was sunbed use >10 times before age 30 years, which was related to all three skin cancers.

## Figures and Tables

**Table 1:** Selected covariates, associations with four ultraviolet radiation indicators as outcomes.

Covariates^∗§^	Sunbed use, 2+ times vs never				Diagnosed with melanocytic nevi				Diagnosed with actinic keratosis				Born in north Sweden			
	*n*	%	OR	CI (95%)	*n*	%	OR	CI (95%)	*n*	%	OR	CI (95%)	*n*	%	OR	CI (95%)
Age 30–66 years^†^	19,946	61.9	**0.91**	**0.91–0.92**	4,614	13.2	**0.97**	**0.97–0.97**	1,649	4.7	**1.12**	**1.11–1.13**	3,476	10.0	**1.03**	**1.03–1.03**
Sex, male ref	6,258	46.2	1.00	Ref	1,393	9.4	1.00	Ref	579	3.9	1.00	Ref	1,384	9.3	1.00	Ref
Female	13,688	73.3	**3.96**	**3.74–4.19**	3,221	16.1	**1.40**	**1.29–1.51**	1,070	5.3	1.03	0.91–1.16	2,092	10.5	1.03	0.95–1.12

Lifestyle factors, physical activity in preceding 12 months																
*At work, low*	13,823	63.2	1.00	Ref	3,233	14.0	1.00	Ref	1,054	4.6	1.00	Ref	2,426	10.5	1.00	Ref
Medium	4,474	59.9	**0.93**	**0.87–0.99**	992	12.6	**0.89**	**0.82–0.97**	459	5.8	0.99	0.87–1.12	761	9.6	**0.91**	**0.83–1.00**
High	1,518	57.8	**0.86**	**0.78–0.94**	249	9.0	**0.79**	**0.68–0.91**	83	3.0	0.83	0.65–1.06	190	6.8	**0.76**	**0.65–0.89**

*Sedentary, low*	9,909	67.6	1.00	Ref	2,201	14.3	1.00	Ref	711	4.6	1.00	Ref	1,535	10.0	1.00	Ref
Medium	7,790	57.9	**0.81**	**0.77–0.86**	1,803	12.6	0.99	0.92–1.06	724	5.1	0.94	0.83–1.05	1,461	10.3	1.02	0.94–1.10
High	2,178	54.7	**0.72**	**0.66–0.78**	492	11.7	0.91	0.81–1.02	171	4.0	**0.81**	**0.67–0.98**	387	9.2	0.90	0.80–1.02

*Walking/cycling, low*	5,841	63.6	1.00	Ref	1,216	12.6	1.00	Ref	353	3.6	1.00	Ref	943	9.7	1.00	Ref
Medium	11,915	62.2	**0.91**	**0.85–0.96**	2,821	13.9	1.04	0.97–1.13	1,020	5.0	**1.16**	**1.02–1.33**	2,142	10.6	1.03	0.94–1.11
High	2,030	56.2	**0.81**	**0.74–0.89**	448	11.8	0.97	0.85–1.10	225	5.9	**1.35**	**1.12–1.62**	296	7.8	**0.77**	**0.67–0.89**

*Binge drinking, never*	6,188	60.7	1.00	Ref	1,648	15.0	1.00	Ref	669	6.1	1.00	Ref	1,151	10.5	1.00	Ref
Sometimes	11,126	65.2	**1.44**	**1.35–1.53**	2,408	13.1	0.96	0.89–1.04	734	4.0	0.98	0.87,1.10	1,875	10.2	1.07	0.99–1.16
Regularly	1,486	52.9	**1.47**	**1.32–1.62**	276	9.0	**0.80**	**0.69–0.93**	140	4.5	1.02	0.83,1.25	272	8.8	0.92	0.80–1.07

*Body shape*, normal weight, short	2,752	63.5	1.00	Ref	704	15.1	1.00	Ref	256	5.5	1.00	Ref	488	10.5	1.00	Ref
Normal weight, medium height	4,127	66.7	**1.10**	**1.01–1.21**	1,014	15.1	0.95	0.85–1.07	389	5.8	1.12	0.94–1.33	705	10.5	0.98	0.87–1.11
Normal weight, tall	4,590	68.6	1.08	0.98–1.18	1,205	16.7	1.04	0.93–1.16	350	4.9	0.95	0.80–1.14	686	9.5	0.89	0.78–1.00
Overweight, short	2,323	54.0	1.01	0.92–1.12	448	9.6	**0.77**	**0.67–0.88**	207	4.5	**0.80**	**0.66–0.98**	482	10.4	0.99	0.87–1.14
Overweight, medium height	3,054	56.2	1.04	0.94–1.14	596	10.2	**0.80**	**0.71–0.91**	230	3.9	**0.77**	**0.63–0.94**	598	10.2	0.99	0.87–1.13
Overweight, tall	2,855	58.9	1.08	0.98–1.19	605	11.5	**0.87**	**0.77–0.99**	195	3.7	**0.71**	**0.58–0.87**	470	8.9	**0.84**	**0.73–0.96**

*Social factors, education level, low*	942	45.1	1.00	Ref	168	7.3	1.00	Ref	100	4.4	1.00	Ref	145	6.3	1.00	Ref
Medium	7,254	61.1	**1.27**	**1.14–1.41**	1,447	11.3	**1.25**	**1.05–1.49**	594	4.6	1.17	0.93–1.48	1,100	8.6	**1.46**	**1.22–1.75**
High	11,746	64.4	1.00	0.89–1.11	2,997	15.2	**1.47**	**1.24–1.76**	955	4.8	**1.32**	**1.05–1.66**	2,230	11.3	**2.14**	**1.78–2.56**

*Type of housing, rental*	3,007	58.2	1.00	Ref	615	11.2	1.00	Ref	193	3.5	1.00	Ref	567	10.4	1.00	Ref
Type of housing, condominium	5,874	60.0	1.03	0.95–1.11	1,500	14.5	**1.20**	**1.08–1.34**	507	4.9	**1.30**	**1.09–1.56**	1,079	10.4	0.92	0.83–1.03
Type of housing, own home	10,744	64.4	**1.35**	**1.26–1.46**	2,331	13.3	1.09	0.98–1.21	894	5.1	**1.43**	**1.21–1.69**	1,693	9.6	**0.82**	**0.74–0.91**
Type of housing, other	242	53.8	**0.71**	**0.56–0.89**	51	10.7	1.04	0.76–1.42	10	2.1	0.81	0.42–1.58	48	10.0	1.12	0.81–1.53
Diabetes, yes, ref is no	418	38.5	**0.71**	**0.62–0.82**	110	9.4	1.02	0.82–1.26	55	4.7	**0.74**	**0.55–0.99**	113	9.7	0.94	0.77–1.15

*Skin diseases, yes, ref is no*																
Rosacea	627	73.0	**1.31**	**1.10–1.56**	291	31.8	**2.11**	**1.80–2.47**	123	13.4	**2.28**	**1.82–2.85**	74	8.1	0.79	0.62–1.01
Other disorders of pigmentation (mostly solar lentigo)	560	68.0	**1.31**	**1.10–1.55**	395	44.8	**3.17**	**2.72–3.70**	232	26.3	**3.32**	**2.77–3.99**	85	9.6	0.89	0.70–1.12
Warts	138	56.1	0.90	0.68–1.21	89	33.5	**2.09**	**1.56–2.80**	48	18.0	**2.22**	**1.54–3.19**	24	9.0	0.90	0.59–1.37
Atopic eczema	404	72.1	1.09	0.88–1.35	135	22.2	**1.27**	**1.02–1.57**	37	6.1	0.91	0.62–1.32	54	8.9	0.96	0.72–1.27
Other eczema	1,815	64.6	1.08	0.98–1.19	671	21.9	**1.46**	**1.31–1.61**	346	11.3	**2.00**	**1.74–2.30**	306	10.0	0.98	0.86–1.12
Seborrheic keratosis	1,283	55.5	0.98	0.88–1.08	1,004	40.4	**5.17**	**4.69–5.71**	437	17.6	**1.96**	**1.71–2.25**	262	10.5	0.99	0.86–1.15

CI, confidence interval; OR, odds ratio; MN, melanocytic nevi; AK, actinic keratosis; ^∗^Adjusted for all other variables in Table 1 and Supplement II. ^§^Values for heredity and ultraviolet radiation indicators are in Supplement II due to lack of space. ^†^Since everyone is in the same age category, 30–66 years, this row shows both the OR when a person becomes one year older and the prevalence of the UVR indicators in the study population. Bold indicates *P* values <0.05. Variables that were tested but insignificant and are not in Table 1: smoking, consumption of vegetables, consumption of fruits and berries, had an influenza vaccine within the preceding 12 months, having had an organ transplant, diagnosed with certain disorders involving the immune system, herpes zoster, diagnosed with psoriasis, diagnosed with vitiligo.

**Table 2:** Ultraviolet radiation indicators and other exposures, associations with basal cell carcinoma, squamous cell carcinoma, and cutaneous malignant melanoma as outcomes.

Exposures ^∗§^	Basal cell carcinoma			Squamous cell carcinoma^∗∗^			Cutaneous malignant melanoma^∗∗^		
	*n* (%)	OR	CI (95%)	*n* (%)	OR	CI (95%)	*n* (%)	OR	CI 95%
Age^†^	1,164 (3.3)	**1.09**	**1.08–1.10**	203 (0.6)	**1.14**	**1.11–1.17**	249 (0.7)	**1.04**	**1.03–1.06**
Sex, male ref	481 (3.2)	1.00	Ref	84 (0.6)	1.00	Ref	82 (0.6)	1.00	Ref
Female	683 (3.4)	**0.82**	**0.71–0.94**	119 (0.6)	0.87	0.64–1.20	167 (0.8)	1.30	0.97–1.74
Sunbed use before age 30 years, never	458 (3.7)	1.00	Ref	96 (0.8)	1.00	Ref	90 (0.7)	1.00	Ref
Once	48 (2.9)	1.09	0.79–1.50	14 (0.8)	**1.81**	**1.01–3.26**	14 (0.8)	1.47	0.82–2.61
2–10 times	253 (3.2)	**1.35**	**1.14–1.60**	39 (0.5)	1.14	0.77–1.69	52 (0.7)	1.21	0.84–1.73
>10 times	380 (3.2)	**1.74**	**1.46–2.06**	53 (0.4)	**1.66**	**1.12–2.47**	87 (0.7)	**1.57**	**1.11–2.22**
Born in north Sweden	123 (3.5)	1.00	Ref	20 (0.6)	1.00	Ref	18 (0.5)	1.00	Ref
Mid-Sweden	830 (3.2)	1.01	0.83–1.24	147 (0.6)	1.21	0.75–1.96	177 (0.7)	1.47	0.90–2.41
South Sweden	211 (3.9)	1.16	0.91–1.47	36 (0.7)	1.26	0.72–2.21	54 (1.0)	**2.17**	**1.26–3.72**
Diagnosed with AK, yes, ref is no	322 (19.5)	**4.06**	**3.46–4.76**	94 (5.7)	—	—		**1.85**	**1.24–2.76**
Diagnosed with MN, yes, ref is no	353 (7.7)	**2.25**	**1.94–2.62**	64 (1.4)	**1.91**	**1.35–2.71**		—	—
History, heredity, yes, ref is no									
Had BCC	—	—	—	67 (5.8)	**6.51**	**4.71–9.00**		**2.72**	**1.82–4.06**
Had SCC	67 (33.0)	**4.12**	**2.93–5.78**	—	—	—		0.99	0.38–2.57
Had CMM	34 (13.7)	**1.91**	**1.26–2.90**	5 (2.0)	0.97	0.37–2.56	—	—	—
Any parent had BCC	273 (5.5)	**1.88**	**1.61–2.20**	35 (0.7)	1.11	0.74–1.65		0.86	0.58–1.27
Any parent had SCC	163 (5.7)	1.08	0.89–1.30	33 (1.1)	**1.51**	**1.01–2.25**		0.83	0.52–1.32
Any parent had CMM	59 (5.8)	**1.34**	**1.00–1.79**	12 (1.2)	1.44	0.77–2.69		**1.80**	**1.02–3.20**
Physical activity during the preceding 12 months									
At work, low	767 (3.3)	1.00	Ref	138 (0.6)	1.00	Ref	152 (0.7)	1.00	Ref
Medium	296 (3.8)	0.97	0.83–1.12	54 (0.7)	0.86	0.62–1.21	67 (0.8)	1.09	0.81–1.47
High	67 (2.4)	0.89	0.68–1.16	11 (0.4)	0.89	0.47–1.69	19 (0.7)	1.17	0.71–1.94
Sedentary, low	507 (3.3)	1.00	Ref	79 (0.5)	1.00	Ref	101 (0.7)	1.00	Ref
Medium	509 (3.6)	0.94	0.82–1.08	102 (0.7)	1.09	0.80–1.49	114 (0.8)	1.13	0.86–1.48
High	122 (2.9)	0.84	0.68–1.04	20 (0.5)	0.77	0.46–1.28	24 (0.6)	0.88	0.56–1.40
Walking/cycling, low	297 (3.1)	1.00	Ref	45 (0.5)	1.00	Ref	62 (0.6)	1.00	Ref
Medium	682 (3.4)	0.93	0.80–1.08	125 (0.6)	1.11	0.78–1.59	145 (0.7)	1.03	0.76–1.40
High	154 (4.0)	1.07	0.87–1.33	31 (0.8)	1.23	0.75–2.00	33 (0.9)	1.09	0.70–1.70
Binge drinking, never	450 (4.1)	1.00	Ref	85 (0.8)	1.00	Ref	106 (1.0)	1.00	Ref
Sometimes	553 (3.0)	0.93	0.81–1.07	84 (0.5)	0.87	0.63–1.21	107 (0.6)	**0.74**	**0.55–0.98**
Regularly	94 (3.1)	0.83	0.65–1.06	18 (0.6)	1.01	0.58–1.74	16 (0.5)	0.59	0.34–1.02
Normal weight, short	152 (3.3)	1.00	Ref	20 (0.4)	1.00	Ref	38 (0.8)	1.00	Ref
Normal weight, medium height	260 (3.9)	1.18	0.95–1.46	52 (0.8)	**1.76**	**1.03–2.98**	41 (0.6)	0.78	0.50–1.23
Normal weight, tall	251 (3.5)	1.13	0.91–1.40	47 (0.7)	1.69	0.99–2.89	45 (0.6)	0.85	0.55–1.33
Overweight, short	119 (2.6)	**0.77**	**0.59–0.99**	23 (0.5)	1.14	0.62–2.12	29 (0.6)	0.84	0.51–1.37
Overweight, medium height	183 (3.1)	1.02	0.81–1.28	29 (0.5)	1.23	0.68–2.21	42 (0.7)	1.02	0.65–1.61
Overweight, tall	182 (3.4)	1.13	0.90–1.42	28 (0.5)	1.29	0.71–2.33	52 (1.0)	1.44	0.94–2.22
Social factors^§^									
Diabetes, yes, ref is no	32 (2.7)	**0.66**	**0.45–0.95**	6 (0.5)	0.67	0.29–1.54	8 (0.7)	0.79	0.38–1.62
Skin diseases, yes, ref is no									
Rosacea	61 (6.7)	1.32	0.98–1.77	14 (1.5)	**1.92**	**1.06–3.48**	7 (0.8)	0.74	0.34–1.61
Other disorders of pigmentation (mostly solar lentigo)	73 (8.3)	0.87	0.66–1.14	18 (2.0)	1.43	0.84–2.43	11 (1.2)	0.85	0.45–1.60
Warts	23 (8.6)	1.22	0.75–1.99	6 (2.3)	1.94	0.82–4.60	6 (2.3)	1.93	0.83–4.50
Atopic eczema	19 (3.1)	0.89	0.54–1.44	^‡^	0.51	0.12–2.20	6 (1.0)	1.37	0.59–3.18
Other eczema	168 (5.5)	1.14	0.95–1.38	32 (1.0)	1.12	0.74–1.69	30 (1.0)	1.01	0.67–1.51
Seborrheic keratosis	231 (9.3)	**1.31**	**1.10–1.56**	55 (2.2)	**1.67**	**1.17–2.39**	49 (2.0)	**2.06**	**1.46–2.90**

CI, confidence interval; OR, odds ratio; UVR, ultraviolet radiation; AK, actinic keratosis; MN, melanocytic nevi; BCC, basal cell carcinoma; SCC, squamous cell carcinoma; CMM, cutaneous malignant melanoma. ^∗∗^Melanoma in situ and SCC in situ were excluded. ^∗^Adjusted for all other variables in Table 2 and Supplement III. ^§^Values for social factors are in Supplement III due to lack of space. ^†^Since everyone is in the same age category, 30–66 years, this row shows both the OR when a person becomes one year older and the prevalence of the skin cancers in the study population. ^‡^Too few cases, *n* < 5. Bold indicates *P* values <0.05.

**Table 3:** Descriptive data on the study population, ultraviolet radiation indicators, and skin cancer, Stockholm County 2014.

Variable	All		Females		Males	
	*n*	(%)	*n*	(%)	*n*	(%)
All	34,841	(100.0)	20,010	(100.0)	14,831	(100.0)
Age 30–49 years	16,522	(47.4)	9,776	(48.9)	6,746	(45.5)
Age 50–66 years	18,319	(52.6)	10,234	(51.1)	8,085	(54.5)

UVR indicators						
*Sunbed use before age 30 years*						
Never	12,281	(35.2)	4,989	(24.9)	7,292	(49.2)
Once	1,676	(4.8)	849	(4.2)	827	(5.6)
2–9 times	7,938	(22.8)	4,755	(23.8)	3,183	(21.5)
>10 times	12,008	(34.5)	8,933	(44.6)	3,075	(20.7)
Missing	938	(2.7)	484	(2.4)	454	(3.1)

*Diagnosed with melanocytic nevi*						
No	30,227	(86.8)	16,789	(83.9)	13,438	(90.6)
Yes	4,614	(13.2)	3,221	(16.1)	1,393	(9.4)

*Diagnosed with actinic keratosis*						
No	33,192	(95.3)	18,940	(94.7)	14,252	(96.1)
Yes	1,649	(4.7)	1,070	(5.3)	579	(3.9)

*Latitude of birthplace*						
Born in south Sweden	5,478	(15.7)	3,189	(15.9)	2,289	(15.4)
Born in mid-Sweden^∗^	25,885	(74.3)	14,728	(73.6)	11,157	(75.2)
Born in north Sweden	3,476	(10.0)	2,092	(10.5)	1,384	(9.3)

Skin cancers						
*Basal cell carcinoma*						
No	33,677	(96.7)	19,327	(96.6)	14,350	(96.8)
Yes	1,164	(3.3)	683	(3.4)	481	(3.2)

*Squamous cell carcinoma* ^∗∗^						
No	34,638	(99.4)	19,891	(99.4)	14,747	(99.4)
Yes	203	(0.6)	119	(0.6)	84	(0.6)

*Cutaneous malignant melanoma* ^∗∗^						
No	34,592	(99.3)	19,843	(99.2)	14,749	(99.4)
Yes	249	(0.7)	167	(0.8)	82	(0.6)

UVR, ultraviolet radiation. ^∗^The region where the study took place. Two persons with missing values on region of birth are not included in the table ^∗∗^Melanoma in situ and squamous cell carcinoma in situ were excluded.

## Data Availability

The data used to support the findings of this study are restricted by the Swedish Ethical Review Authority in order to protect patient privacy. Data are available from Henrik Dal, henrik.dal@regionstockholm.se for researchers who meet the criteria for access to confidential data.

## Supplementary Materials

Supplementary Materials Supplement Table I. Description of the variables in the article. Supplement Table II. Selected covariates, associations with four ultraviolet radiation indicators as outcomes. Supplementary Table III. Social factors and the associations with basal cell carcinoma, squamous cell carcinoma, and cutaneous malignant melanoma as outcomes.
