# Supplementary material for: Analysis of the Stockholm Public Health Cohort: Exploring How Ultraviolet Radiation and Other Factors Associate with Skin Cancer
Source: J Skin Cancer. 2024 Oct 3;2024:7142055. doi: 10.1155/2024/7142055 (PMC11480963; doi:10.1155/2024/7142055)
Supplement: Supplementary Materials — Supplement Table I. Description of the variables in the article. Supplement Table II. Selected covariates, associations with four ultraviolet radiation indicators as outcomes. Supplementary Table III. Social factors and the associations with basal cell carcinoma, squamous cell carcinoma, and cutaneous malignant melanoma as outcomes. [file 7142055.f1.docx]

# Supplementary tables

**Supplement Table I. Description of the variables in the article.**

| Outcome | Measure | Source | Values/Codes |
| --- | --- | --- | --- |
| Basal cell carcinoma | All rows | Swedish Basal Carcinoma Register 2004–2018 |  |
| Squamous cell carcinoma (SCC) | ICD-7 | Swedish Cancer Registry 1958–2017 | 191 (ICD-7)  SCC in situ excluded. |
| Cutaneous malignant melanoma  (CMM) | ICD-7, malignant | Swedish Cancer Registry 1958–2017 | 190 (ICD-7) Melanoma in situ excluded. |
| Covariates | **Measure** | **Source** |  |
| UV exposure | Sunbed use before age 30 years | Stockholm Public Health Cohort 2014 | Never, Once, 2–10 times, >10 times |
| UV exposure | Latitude of birthplace in Sweden | Total Population Register | South/mid/north |
| UV exposure | Diagnosed with actinic keratosis | The National Patient Register | Y/N, L57 (ICD-10) |
| UV exposure | Diagnosed with melanocytic nevi | National Patient Register | Y/N, D22 (ICD-10) |
| Age | Age in year 2014 | Register of Total Population | 30–66 years |
| Sex | Sex at birth | Register of Total Population | Male/female |
| Skin type, hair colour etc. | Born in Sweden, part of selecting the study population | Register of Total Population | Yes |
| Heredity/UVR exposure | Parental skin cancer | Swedish Basal Carcinoma Register /  Swedish Cancer Registry | BCC: Any registered cancer in the BCC register  SCC: 191 (ICD7) in the Cancer registry  CMM: 190 (ICD7, malignant) in the Cancer registry. Melanoma in situ and SCC in situ were excluded. |
| Body shape | *Height*  Short  Male <178 cm  Female <164 cm  Average  Male 178–183 cm  Female 164–169 cm  Tall  Male >183 cm  Female >169 cm  *Body mass index* | Stockholm Public Health Cohort 2014 | Six groups. Two body mass index categories: 1) normal (incl. underweight) or 2) overweight (incl. obese). Male and female participants were separately divided into three height categories: 1) short, 2) medium, 3) tall, with about the same number of male or female participants in each group. |
| Lifestyle factors |  |  |  |
| Dietary factors | Two dietary factors (vegetables, fruits/berries) | Stockholm Public Health Cohort 2014 | 14 frequency categories from Never to 4+ times per day were collapsed into three categories: 1) Never / Three times per month, 2) 1-6 times per week, 3) Once a day or more. |
| Physical activity | Three factors about physical activity (at work, sedentary behavior, walking/cycling) | Stockholm Public Health Cohort 2014 | 6-7 frequency categories, depending on the type of activity, were collapsed into three: 1) Low, 2) Medium 3) High |
| Smoking | Had ever smoked for at least 6 months | Stockholm Public Health Cohort 2014 | Y/N |
| Alcohol consumption | Binge drinking during preceding 12 months | Stockholm Public Health Cohort 2014 | 7 frequency categories of binge drinking from Never to at least 5 days per week, were collapsed into three: 1) Never, 2) Sometimes (2-3 times per month) 3) Regularly (Once a week or more) |
| Social factors |  |  |  |
| Education level | Highest attained education level | Longitudinal Integrated Database for Health Insurance and Labor Market Studies 2014 | Three levels: 1) low – grade school, 2) average – high school, 3) high – college/university |
| Type of residence |  | Stockholm Public Health Cohort 2014 | 7 categories collapsed into 4 (rental, condominium, own home, other) |
| Medical factors |  |  |  |
| Herpes zoster | Diagnosed with herpes zoster | National Patient Register | Y/N, B02 (ICD-10) |
| Warts | Diagnosed with virus warts | National Patient Register | Y/N, B07 (ICD-10) |
| Atopic eczema | Diagnosed with atopic eczema | National Patient Register | Y/N, L20 (ICD-10) |
| Other eczema | Diagnosed with other eczema than atopic eczema | National Patient Register | Y/N, L21-L30 (ICD-10) |
| Rosacea | Diagnosed with rosacea | National Patient Register | Y/N, L71 (ICD-10) |
| Vitiligo | Diagnosed with vitiligo | National Patient Register | Y/N, L80 (ICD-10) |
| Seborrheic keratosis | Diagnosed with seborrheic keratosis | National Patient Register | Y/N, L82 (ICD-10) |
| Psoriasis | Had been diagnosed with psoriasis by MD | Stockholm Public Health Cohort 2014 | Y/N |
| Diabetes | Had been diagnosed with diabetes by MD | Stockholm Public Health Cohort 2014 | Y/N |
| Pigment disorders | Diagnosed with other pigment disorders (solar lentigo) | National Patient Register | Y/N, L81 (ICD-10) |
| Immune disorders | Diagnosed with certain disorders involving the immune system | National Patient Register | Y/N, D80-89 (ICD-10) |
| Organ transplant | Had organ transplant | National Patient Register | Y/N, Z94 (ICD-10) |
| Influenza vaccine | Had an influenza vaccine within preceding 12 months | Stockholm Public Health Cohort 2014 | Y/N |

**Supplement Table II. Selected covariates, associations with four ultraviolet radiation indicators as outcomes.**

| Covariates* | Sunbed use, 2+ times vs never | | | | Diagnosed with melanocytic nevi | | | | Diagnosed with actinic keratosis | | | | Born in north Sweden | | | |
| --- | --- | --- | --- | --- | --- | --- | --- | --- | --- | --- | --- | --- | --- | --- | --- | --- |
|  | n | % | OR | CI (95%) | n | % | OR | CI (95%) | n | % | OR | CI (95%) | n | % | OR | CI (95%) |
| Sunbed use before age 30 years, Never | - | - | - | - | 1,126 | 9.2 | 1.00 | Ref | 699 | 5.7 | 1.00 | Ref | 1,145 | 9.3 | 1.00 | Ref |
| Once | - | - | - | - | 214 | 12.8 | **1.22** | **1.03–1.44** | 79 | 4.7 | **1.29** | **1.00–1.67** | 138 | 8.2 | 1.01 | 0.83–1.21 |
| 2–10 times | - | - | - | - | 1,117 | 14.1 | **1.32** | **1.20–1.45** | 336 | 4.2 | **1.20** | **1.03–1.40** | 786 | 9.9 | **1.25** | **1.13–1.38** |
| >10 times | - | - | - | - | 2,043 | 17.0 | **1.51** | **1.38–1.65** | 489 | 4.1 | **1.62** | **1.39–1.88** | 1,315 | 11.0 | **1.51** | **1.37–1.67** |
| Born in north Sweden | 2,101 | 64.7 | 1.00 | Ref | 385 | 11.1 | 1.00 | Ref | 192 | 5.5 | 1.00 | Ref | - | - | - | - |
| Mid-Sweden | 14,543 | 60.9 | **0.71** | **0.65–0.78** | 3,419 | 13.2 | **1.26** | **1.12–1.42** | 1169 | 4.5 | 0.91 | 0.77–1.07 | - | - | - | - |
| South Sweden | 3,301 | 64.8 | **0.87** | **0.78–0.96** | 810 | 14.8 | **1.30** | **1.14–1.50** | 287 | 5.2 | 1.01 | 0.83–1.24 | - | - | - | - |
| Diagnosed with AK, Yes, ref is No | 825 | 54.1 | 1.06 | 0.94–1.20 | 525 | 31.8 | **2.40** | **2.12–2.73** | - | - | - | - | 385 | 8.3 | 1.09 | 0.93–1.29 |
| Diagnosed with MN, Yes, ref is No | 3,160 | 73.7 | **1.43** | **1.31–1.56** | - | - | - | - | 525 | 11.4 | **2.54** | **2.24–2.89** | 192 | 11.7 | **0.79** | **0.70–0.89** |
| Heredity, Yes, ref is No |  |  |  |  |  |  |  |  |  |  |  |  |  |  |  |  |
| Any parent had BCC | 3,090 | 67.2 | **1.15** | **1.07–1.25** | 870 | 17.5 | **1.28** | **1.17–1.40** | 298 | 6.0 | **1.39** | **1.20–1.60** | 396 | 8.0 | **0.78** | **0.70–0.88** |
| Any parent had SCC** | 1,585 | 59.5 | 1.08 | 0.98–1.18 | 465 | 16.2 | 1.09 | 0.97–1.23 | 260 | 9.1 | **1.53** | **1.31–1.78** | 231 | 8.1 | **0.75** | **0.65–0.87** |
| Any parent had CMM** | 611 | 64.8 | 1.08 | 0.92–1.26 | 262 | 25.6 | **2.20** | **1.88–2.57** | 63 | 6.1 | 1.01 | 0.76–1.35 | 71 | 6.9 | **0.70** | **0.54–0.89** |

CI, confidence interval; OR, odds ratio; AK, actinic keratosis; MN, melanocytic nevi; BCC, basal cell carcinoma; SCC, squamous cell carcinoma; CMM, cutaneous malignant melanoma. ** Melanoma in situ and SCC in situ were excluded. *Adjusted for all other variables in Table I and in Supplement II. Bold indicates P-values <0.05.

**Supplementary Table III. Social factors and the associations with basal cell carcinoma, squamous cell carcinoma and cutaneous malignant melanoma as outcomes.**

| Exposures* | Basal cell carcinoma | | | Squamous cell carcinoma** | | | Cutaneous malignant melanoma** | | |
| --- | --- | --- | --- | --- | --- | --- | --- | --- | --- |
|  | n (%) | OR | CI (95%) | n (%) | OR | CI (95%) | n (%) | OR | CI (95%) |
| Education level, low | 84 (3.7) | 1.00 | Ref | 15 (0.7) | 1.00 | Ref | 22 (1.0) | 1.00 | Ref |
| Medium | 428 (3.3) | 0.98 | 0.76–1.26 | 69 (0.5) | 1.04 | 0.58–1.85 | 97 (0.8) | 0.86 | 0.54–1.38 |
| High | 652 (3.3) | 0.98 | 0.76–1.26 | 119 (0.6) | 1.28 | 0.72–2.26 | 130 (0.7) | 0.80 | 0.49–1.29 |
| Type of housing, rental | 156 (2.8) | 1.00 | Ref | 32 (0.6) | 1.00 | Ref | 37 (0.7) | 1.00 | Ref |
| Type of housing, condominium | 350 (3.4) | 1.04 | 0.85–1.27 | 62 (0.6) | 0.92 | 0.59–1.43 | 79 (0.8) | 1.17 | 0.78–1.75 |
| Type of housing, own home | 629 (3.6) | 1.10 | 0.91–1.33 | 107 (0.6) | 0.99 | 0.66–1.50 | 120 (0.7) | 1.06 | 0.72–1.55 |
| Type of housing, other | 5 (1.0) | 0.49 | 0.20–1.21 | ‡ | 0.61 | 0.08–4.51 | ‡ | 1.71 | 0.60–4.85 |

CI, confidence interval; OR, odds ratio; *Adjusted for all other variables in Table II and in Supplement III. SCC, squamous cell carcinoma. **Melanoma in situ and SCC in situ were excluded. ‡Too few cases, n<5.
